# Association of serum cortisol and cortisone levels and risk of recurrence after endocrine treatment in breast cancer

**DOI:** 10.1007/s10238-023-01109-x

**Published:** 2023-07-03

**Authors:** Feng Wang, Guro F. Giskeødegård, Sissel Skarra, Monica J. Engstrøm, Lars Hagen, Jürgen Geisler, Tomi S. Mikkola, Matti J. Tikkanen, Julia Debik, Randi J. Reidunsdatter, Tone F. Bathen

**Affiliations:** 1 Department of Circulation and Medical Imaging, Norwegian University of Science and Technology, NTNU, Trondheim, Norway; 2 Department of Breast and Endocrine Surgery, St. Olavs Hospital, Trondheim University Hospital, Trondheim, Norway; 3 K.G. Jebsen Center for Genetic Epidemiology, Department of Public Health and Nursing, Norwegian University of Science and Technology, NTNU, Trondheim, Norway; 4 Deprtment of Clinical and Molecular Medicine, Norwegian University of Science and Technology, NTNU, Trondheim, Norway; 5 Clinic of Laboratory Medicine, St. Olavs Hospital, Trondheim, Norway; 6 PROMEC Core Facility for Proteomics and Modomics, Norwegian University of Science and Technology, NTNU, and the Central Norway Regional Health Authority Norway, Trondheim, Norway; 7 Deparment of Oncology, Akershus University Hospital, Lørenskog, Norway; 8 Institute of Clinical Medicine, Faculty of Medicine, University of Oslo, Oslo, Norway; 9 Department of Gynecology, Helsinki University Hospital, Helsinki, Finland; 10 Folkhälsan Research Center, University of Helsinki, Helsinki, Finland; 11 Heart and Lung Center, University of Helsinki and Helsinki University Hospital, Helsinki, Finland

**Keywords:** Breast cancer recurrence, Fatigue, Steroid hormones, Cortisol, Cortisone

## Abstract

Metabolic reprogramming in breast cancer involves changes in steroid hormone synthesis and metabolism. Alterations in estrogen levels in both breast tissue and blood may influence carcinogenesis, breast cancer growth, and response to therapy. Our aim was to examine whether serum steroid hormone concentrations could predict the risk of recurrence and treatment-related fatigue in patients with breast cancer. This study included 66 postmenopausal patients with estrogen receptor-positive breast cancer who underwent surgery, radiotherapy, and adjuvant endocrine treatment. Serum samples were collected at six different time points [before the start of radiotherapy (as baseline), immediately after radiotherapy, and then 3, 6, 12 months, and 7–12 years after radiotherapy]. Serum concentrations of eight steroid hormones (cortisol, cortisone, 17α-hydroxyprogesterone, 17β-estradiol, estrone, androstenedione, testosterone, and progesterone) were measured using a liquid chromatography–tandem mass spectrometry-based method. Breast cancer recurrence was defined as clinically proven relapse/metastatic breast cancer or breast cancer-related death. Fatigue was assessed with the QLQ-C30 questionnaire. Serum steroid hormone concentrations measured before and immediately after radiotherapy differed between relapse and relapse-free patients [(accuracy 68.1%, *p*  = 0.02, and 63.2%, *p*  = 0.03, respectively, partial least squares discriminant analysis (PLS-DA)]. Baseline cortisol levels were lower in patients who relapsed than in those who did not (*p* < 0.05). The Kaplan–Meier analysis showed that patients with high baseline concentrations of cortisol (≥ median) had a significantly lower risk of breast cancer recurrence than patients with low cortisol levels (<median) (*p*  = 0.02). During follow-up, there was a decrease in cortisol and cortisone concentrations in relapse-free patients, whereas these steroid hormones increased in patients who relapsed. In addition, steroid hormone concentrations immediately after radiotherapy were associated with treatment-related fatigue (accuracy of 62.7%, *p*  = 0.03, PLS-DA). However, baseline steroid hormone levels did not predict fatigue at 1 year or at 7–12 years. In conclusion, breast cancer patients with low baseline cortisol levels were more likely to experience recurrence. During follow-up, cortisol and cortisone levels decreased in relapse-free patients but increased in patients with recurrence. Thus, cortisol and cortisone may act as potential biomarkers indicating individual risk of recurrence.

**Supplementary Information:**

The online version contains supplementary material available at 10.1007/s10238-023-01109-x.

## Background

Approximately 70–80% of breast cancers are considered hormone-dependent due to expression of the estrogen receptor (ER) and/or progesterone receptor (PR) [1]. For these breast cancer subtypes, most of which are luminal A and luminal B subtypes, endocrine treatment is the mainstay of systemic treatment for both early and metastatic disease [2]. The introduction of tamoxifen and aromatase inhibitors has significantly improved clinical outcomes for patients with ER positive breast cancer [3]. However, resistance to endocrine treatment occurs in many patients, leading to either local recurrence or metastatic breast cancer during or after adjuvant endocrine therapy or metastatic disease progression during endocrine therapy [3]. Reliable factors indicating a higher risk of breast cancer recurrence are still lacking, although the situation has improved somewhat recently with the use of gene arrays [4]. The discovery of novel biomarkers to predict the risk of recurrence would help to improve clinical decision making for endocrine treatment in the adjuvant or metastatic phase.

The production of estrogens is involved in the complex pathway of cholesterol metabolism, in which glucocorticoids, progestogens, androgens, and estrogens are sequentially formed. The importance of these steroid hormones in the development of breast cancer is previously established [5]. In postmenopausal women, higher levels of endogenous estrogens and androgens are strongly associated with breast cancer risk, especially ER positive breast cancer [6, 7]. It has been shown that among the major estrogen metabolic pathways, the 2-hydroxylation pathway, rather than the competing 16-hydroxylation pathway, was associated with a lower risk of breast cancer in postmenopausal women receiving estrogen [8]. While studies on the serum levels of estrogens in relation to breast cancer risk in premenopausal women have been inconclusive, higher levels of circulating androgen levels are shown to correlate with increased risk of breast cancer. [9, 10] However, only few studies have examined the association between multiple steroid hormones and breast cancer recurrence [11–13].

In breast cancer survivors, fatigue is the most commonly reported symptom affecting function, ability to work, and quality of life [14, 15]. Several biological mechanisms have been proposed to underlie cancer-related fatigue, such as inflammatory dysfunction and alterations in the hypothalamic-pituitary-adrenal (HPA) axis [16], e.g. through alterations in glucocorticoid production and/or decreased sensitivity of the glucocorticoid receptor to hormone ligation [17]. Cortisol is one of the major human glucocorticoids, and increased levels of evening salivary cortisol and higher total salivary cortisol secretion have been found in breast cancer survivors with persistent fatigue [18]. Decreased cognitive dysfunction as a mental component of fatigue may be related to cancer, cancer-related treatment, or cancer-related post-traumatic stress [19]. A better understanding of how cortisol and other steroid hormones is involved in the mechanisms leading to treatment-related fatigue could help identify patients at risk and develop strategies to counteract such adverse events.

Metabolomics can improve our understanding of pathophysiological changes and may be a source of new clinically relevant biomarkers [20, 21]. As a subfield of metabolomics, steroidomics deals with the characterization and quantification of steroids and has been applied in various fields such as doping analysis and the identification of clinically relevant disease biomarkers [22]. Compared to the conventional immunoaffinity-based assays, liquid chromatography–tandem mass spectrometry (LC–MS/MS) and gas chromatography–mass spectrometry (GC–MS) are very sensitive and accurate for simultaneous measurement of steroid hormones in biofluids. Due to the relationship between steroidogenesis and carcinogenesis in breast cancer, mass spectrometry-based steroidomics approaches have recently been introduced into breast cancer research [23], which may play an important role in monitoring disease progression, improving prognosis, and reducing recurrence.

The aim of this study was to examine whether serum steroid hormone concentrations measured after surgery, but before and after radiotherapy, could predict the risk of recurrence and treatment-related fatigue in patients with ER positive breast cancer.

## Methods

### Subjects and study design

Our patient cohort was part of a larger study investigating late effects and health-related quality of life after radiotherapy. Detailed information on the recruitment, follow-up, and compliance of the study has been published previously [23]. Briefly, all patients were diagnosed with invasive, non-metastatic breast carcinoma, had the tumor surgically removed, and were scheduled for postoperative local or locoregional radiotherapy at St. Olav Hospital in Trondheim, Norway, between 2007 and 2008. We included 66 ER positive patients. All patients were postmenopausal and received adjuvant endocrine treatment but no other systemic treatment including chemotherapy. In 2007–2008, the options for adjuvant endocrine treatment for postmenopausal patients in Norway were either monotherapy with tamoxifen, sequential use of tamoxifen followed by an aromatase inhibitor, or aromatase inhibitor monotherapy for 5 years [24]. Serum samples were collected at six time points after breast cancer surgery: before the start of radiotherapy (TP1), immediately after radiotherapy (TP2), and then 3 (TP3), 6 (TP4), and 12 months (TP5) and 7–12 years (TP6) after radiotherapy. Samples were stored at −80 °C until analysis. At each time point, patient-reported outcomes including fatigue were collected using the European Organization for Research and Treatment of Cancer (EORTC) Quality of Life Questionnaire 30 (QLQ-C30).

### Measurement of steroid hormone concentrations in serum

Serum concentrations of nine steroid hormones (17β-estradiol, estrone, 17α-hydroxyprogesterone, cortisol, cortisone, corticosterone, androstenedione, testosterone, and progesterone) were measured using an internally validated LC–MS/MS method. An internal standard mixture containing the nine deuterated steroid hormones (20 µl) was added to 250 µl of serum samples, calibrators, and quality controls. Extraction was performed twice by adding 1 ml of hexane/methyl tert-butyl ether 3:1 vol/vol. The organic phases of the two extracts were mixed and then evaporated to dryness. Samples were dissolved in 50 µl of 50% methanol before injection (10 µl) into the LC–MS/MS.

The LC–MS/MS analysis was performed using an API 5500 triple quadrupole mass spectrometer (AB Sciex, Concord, CA). Peripheral equipment included a Prominence series HPLC system with a binary pump LC-20AD (Shimadzu, Kyoto, Japan). Separation was performed using the following columns: a SunFire C18 column (50 mm × 2.1 mm, 3.5 μm, Waters, Milford, MA) and a Discovery HS F5 HPLC column (100 mm × 2.1 mm, 3 µm, Merck Life Science AS, Norway) and a guard column (Discovery HS F5 SuperGuard column, 20 mm × 2.1 mm, 3 µm, Merck Life Science AS, Norway). The mobile phase was 0.1 mM NH4F in water (A) and 0.1 mM NH4F methanol: water (99:1, vol/vol) (B), with a flow rate of 200 μl/min. The gradient was as follows: 0 min, 40% B; 0–3.5 min, linear increase to 68% B; 3.5–9.5 min, from 68 to 71% B; 9.5–13.5 min, from 71 to 80% B; 13.5–14.5 min, from 80 to 100% B; 14.5–19.1 min, 100% B; and 19.1–21 min, linear decrease to 40% B. Steroid hormones were analyzed by multiple reaction monitoring, which was essentially the same as the method described by Häkkinen et al. [25], but with some modifications. The negative ion mode method included 17β-estradiol, estrone, 17α-hydroxyprogesterone, cortisol, cortisone, and corticosterone, and the positive ion mode method included androstenedione, testosterone, and progesterone. Data were acquired and processed using Analyst software (version 1.7.2, AB Sciex). The limit of detection was the signal-to-noise ratio (S/N) > 3. The lower limit of quantification (LLOQ) was the concentration of the lowest calibrator for each steroid hormone. The percentage of samples with concentrations below the LLOQ was 54.1%, 36.6%, and 20.9% for 17β-estradiol, progesterone, and estrone, respectively, for all samples measured. For five steroid hormones (17α-hydroxyprogesterone, testosterone, androstenedione, cortisol, and cortisone), the values were below the LLOQs in less than 0.1% of the samples. Corticosterone was excluded for further analysis because the results of all samples were below the LLOQ.

### Clinical endpoints

Information on clinical follow-up of up to 15 years was available for these patients. Breast cancer recurrence was defined as clinically proven relapse/metastatic breast cancer or breast cancer-related death during follow-up. Physical fatigue was assessed using the fatigue subscale of the EORTC QLQ-C30 at time points TP1-TP6 [26], while the mental dimension of fatigue was assessed using the cognitive function subscale as a proxy. Response options ranged from 1 (not at all) to 4 (very much), and both subscales were averaged and transformed into a scale of 0 to 100 according to the EORTC scoring manual. Scores above 39 for physical fatigue and below 75 for mental fatigue (reduced cognitive functioning) were considered clinically relevant [27], and the variables were dichotomized accordingly.

### Statistical analysis

Statistical analysis was performed in MATLAB R2021b and with R software. Steroid hormone concentrations whose values were below the lower limit of quantification (LLOQ) were replaced with imputed data obtained by a compositional approach using the R package zComp [28]. Chi-square tests for categorical variables or Wilcoxon signed-rank tests for continuous variables were used to compare clinical parameters between patients with and without recurrence. For comparison of serum steroid hormone concentrations between baseline and the other five time points and between two consecutive time points, Wilcoxon rank tests were performed because the data were not normally distributed.

Linear mixed models (LMM) were applied to determine longitudinal changes in steroid hormones between patient groups. Models were adjusted for BMI and age as potential confounders. Time, patient groups (relapse/nonrelapse or fatigue/nonfatigue), time-group interactions, BMI, and age were included as fixed effects, whereas patient ID was included as a random effect. Multiple-test correction was performed according to the Benjamini–Hochberg procedure, and the adjusted *p* < 0.05 was considered significant. Kaplan–Meier analysis was performed to calculate the probability of survival (breast cancer recurrence, i.e., relapse or breast cancer-specific death) for patients with high (≥ median) and low (< median) baseline levels of steroid hormones (cortisol and cortisone), and the difference between survival curves was tested using the log-rank test.

Partial least square discriminant analysis (PLS-DA) was performed to discriminate between patient groups (recurrence/non-recurrence and fatigue/nonfatigue) on the basis of serum steroid hormone levels at each time point. These analyses were performed using PLS toolbox version 9 in MATLAB R2021b. Models were validated by fivefold cross-validation with 20 iterations, and the mean cross-validated classification results are presented. The significance of the classification was evaluated by permutation tests with 2000 repetitions, and the permutation *p* value was calculated.

## Results

### Patient cohort

The clinicopathologic characteristics of the patients are summarized in Table 1. Body mass index (BMI) was higher in patients with recurrent breast cancer (Wilcoxon signed-rank test, *p* = 0.03), while there was no significant difference in grade, tumor size, and nodal spread. In our patient cohort, 56 patients received sequential use of tamoxifen followed by aromatase inhibitors (53 started tamoxifen during radiotherapy and 3 after radiotherapy), and 10 patients received aromatase inhibitors for 5 years (all started during radiotherapy). The types of endocrine treatment during serum collection at different time points are shown in Supplementary Table 1. The clinical data of ten patients with recurrent breast cancer are shown in Supplementary Table 2. Of these, one patient died within five years (4.8 years) of diagnosis. The number and percentage of patients with physical and mental fatigue are shown in Table 1.

**Table 1:** Clinicopathological characteristics

		Whole cohort	No recurrence	Breast cancer recurrence
		*N*(%)	*N*(%)	*N*(%)
n		66	56	10
Age(median, range)		64.5 (55–89)	66 (55–89)	64 (56–88)
Grade	1	10 (15.2%)	10 (17.9%)	0
	2	38 (57.6%)	31 (55.4%)	7 (70%)
	3	17 (25.8%)	14 (25.0%)	3 (30%)
	Other	1 (1.5%)	1 (1.8%)	0
Tumor size(T)	T1 (< 2 cm)	44 (66.7%)	37 (66.1%)	7 (70%)
	T2 (2–5 cm)	20 (30.3%)	17 (30.4%)	3 (30%)
	T3 (> 5 cm)	0	0	0
	T4 (skin or chest wall)	2 (3.0%)	2 (3.6%)	0
Nodal spread(N)	N0	43 (65.2%)	38 (67.9%)	5 (50%)
	N1	21 (31.8%)	17 (30.4%)	4 (40%)
	N2	2 (3.0%)	1 (1.8%)	1 (10%)
Stage (AJCC)	Stage I	30 (45.5%)	25 (44.6%)	5 (50%)
	Stage IIA	25 (37.9%)	23 (41.1%)	2 (20%)
	Stage IIB	7 (10.6%)	5 (8.9%)	2 (20%)
	Stage IIIA	2 (3.0%)	1 (1.8%)	1 (10%)
	Stage IIIB	2 (3.0%)	2 (3.6%)	0
BMI (median, range)*		25.8 (18.9–38.4)	24.5 (18.9–38.4)	27.8 (24.7–34.4)
Endocrine treatment	Tamoxifen started during RT^#^	53 (80.3%)	44 (78.6%)	9 (90%)
	Tamoxifen after RT	3 (4.5%)	3 (5.3%)	0
	Aromatase inhibitors during RT	10 (15.2%)	9 (16.1%)	1 (10%)
Physical fatigue	TP 1	12 (18.5%)	10 (18.2%)	2 (20%)
	TP 2	25 (38.5%)	22 (40%)	3 (30%)
	TP 3	18 (30.0%)	13 (26.5%)	5 (55.6%)
	TP 4	16 (29.6%)	11 (24.4%)	5 (55.6%)
	TP 5	17 (27.4%)	13 (25%)	4 (40%)
	TP 6	18 (38.3%)	16 (36.4%)	2 (66.7%)
Mental fatigue (reduced cognitive functioning)	TP 1	16 (24.6%)	12 (21.8%)	4 (40.0%)
	TP 2	14 (21.5%)	10 (18.2%)	4 (40.0%)
	TP 3	13 (23.2%)	10 (21.3%)	3 (33.3%)
	TP 4	14 (25.9%)	11 (24.4%)	3 (33.3%)
	TP 5	15 (24.6%)	11(21.6%)	4 (40.0%)
	TP 6	9 (19.1%)	7 (15.9%)	2 (66.7%)

*Significantly different between patients with and without recurrence (*p* = 0.031, Wilcoxon signed-rank test)

^#^RT represents radiotherapy

AJCC, the American Joint Committee on Cancer

BMI, body mass index

### Association of serum steroid hormone levels with breast cancer recurrence

**Fig. 1:**
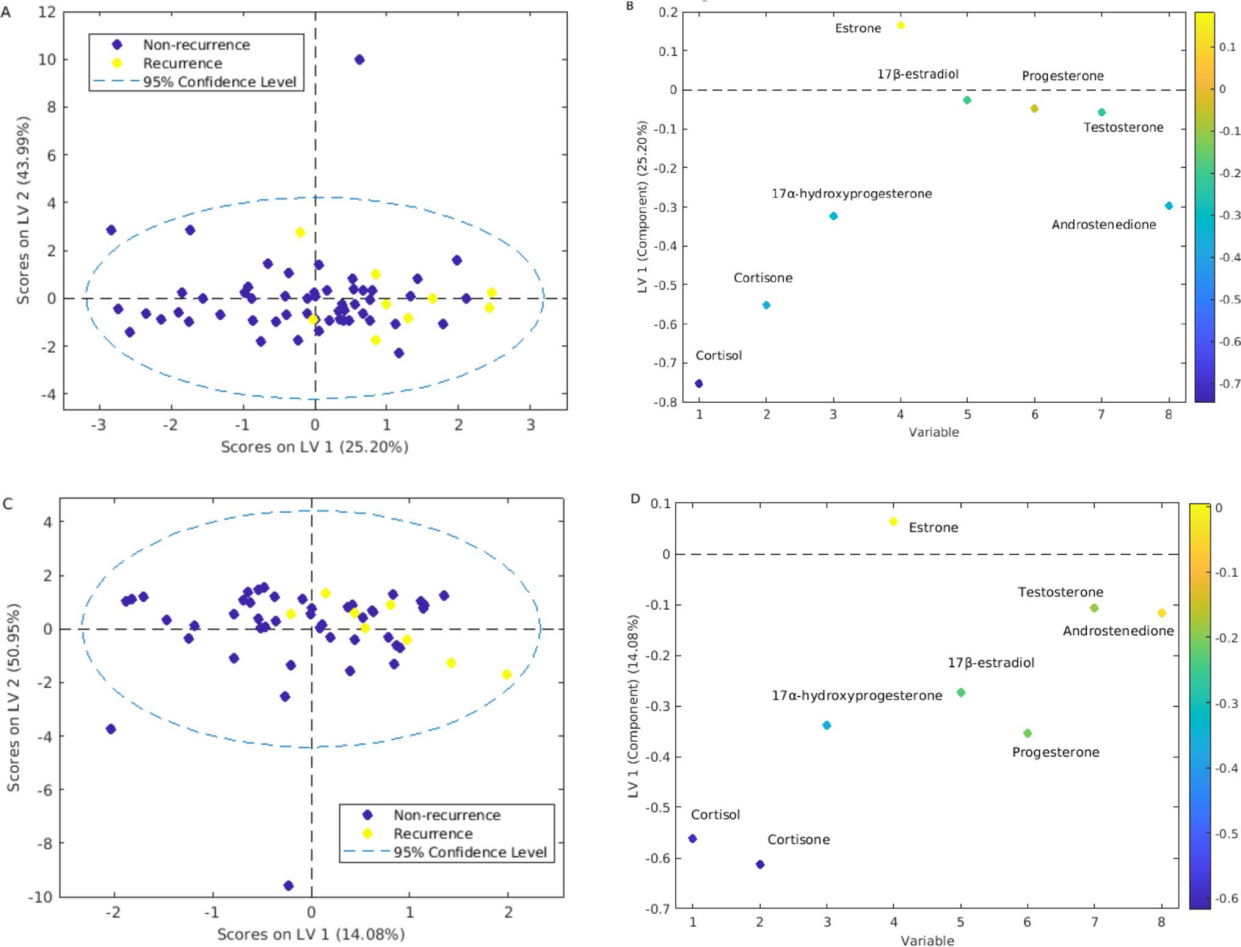
Scores and loadings plots from PLS-DA for prediction of recurrence based on steroid hormone concentrations. **A**, **B** serum steroid hormone levels were measured at TP1; prediction sensitivity: 62.1%, specificity: 74.2%, accuracy = 68.0%, permutation *p* = 0.02. **C**, **D** serum steroid hormone levels were measured at TP2; prediction sensitivity: 55.8%, specificity: 74.6%, accuracy = 63.2%, permutation *p* = 0.03. The color bar in B and D represents weights on LV 1

**Fig. 2:**
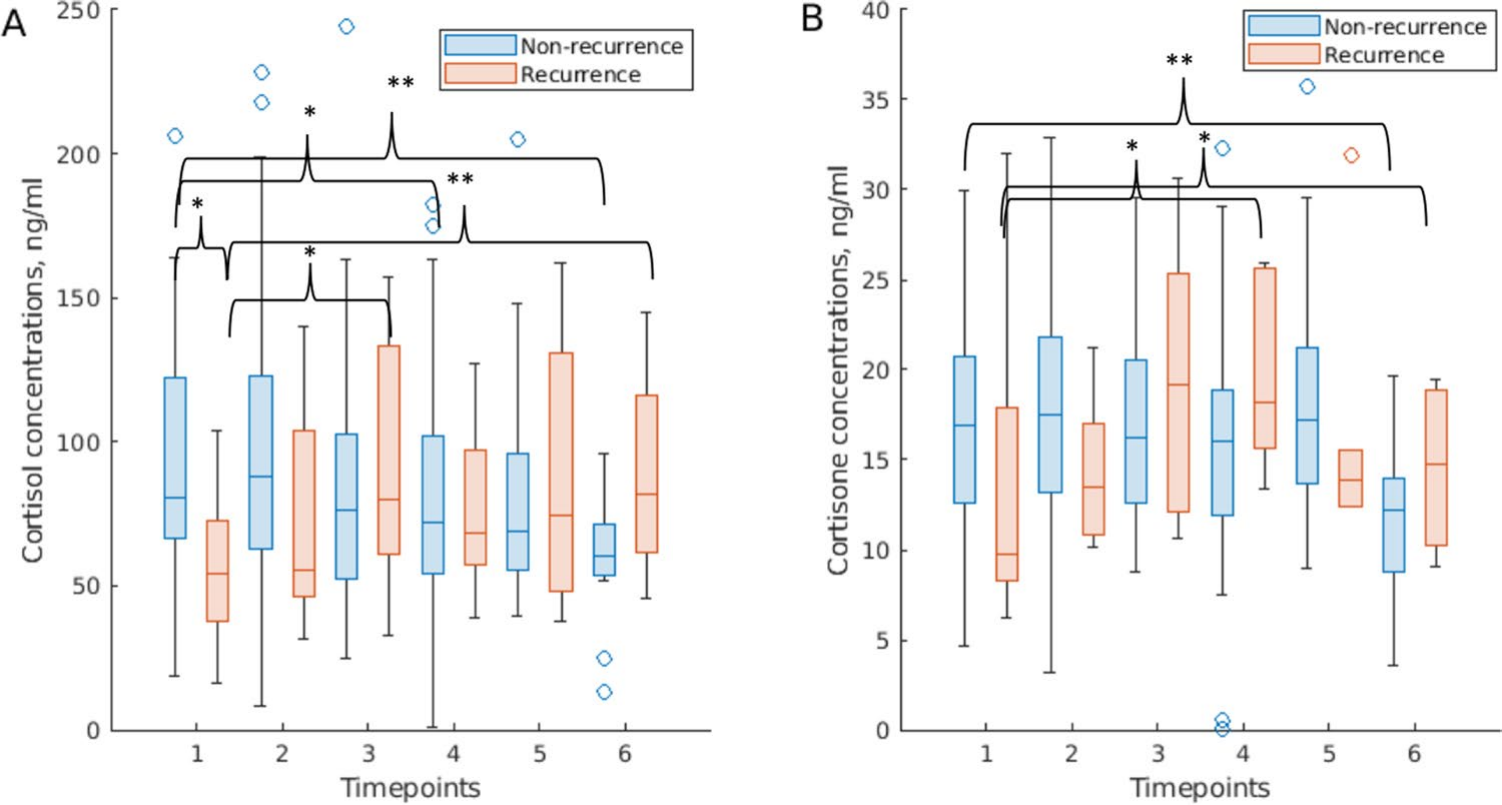
Changes in serum cortisol and cortisone levels among patients with and without recurrence. **A** Cortisol. **B** Cortisone. LMM, models were adjusted for BMI and age as possible confounders, reference coded to TP1 and non-recurrence. **, adjusted *p* ˂0.01, *, adjusted *p* ˂0.05. Compared to baseline levels (TP1), serum levels of cortisol and cortisone decreased in relapse-free patients and increased in patients with recurrent breast cancer

**Fig. 3:**
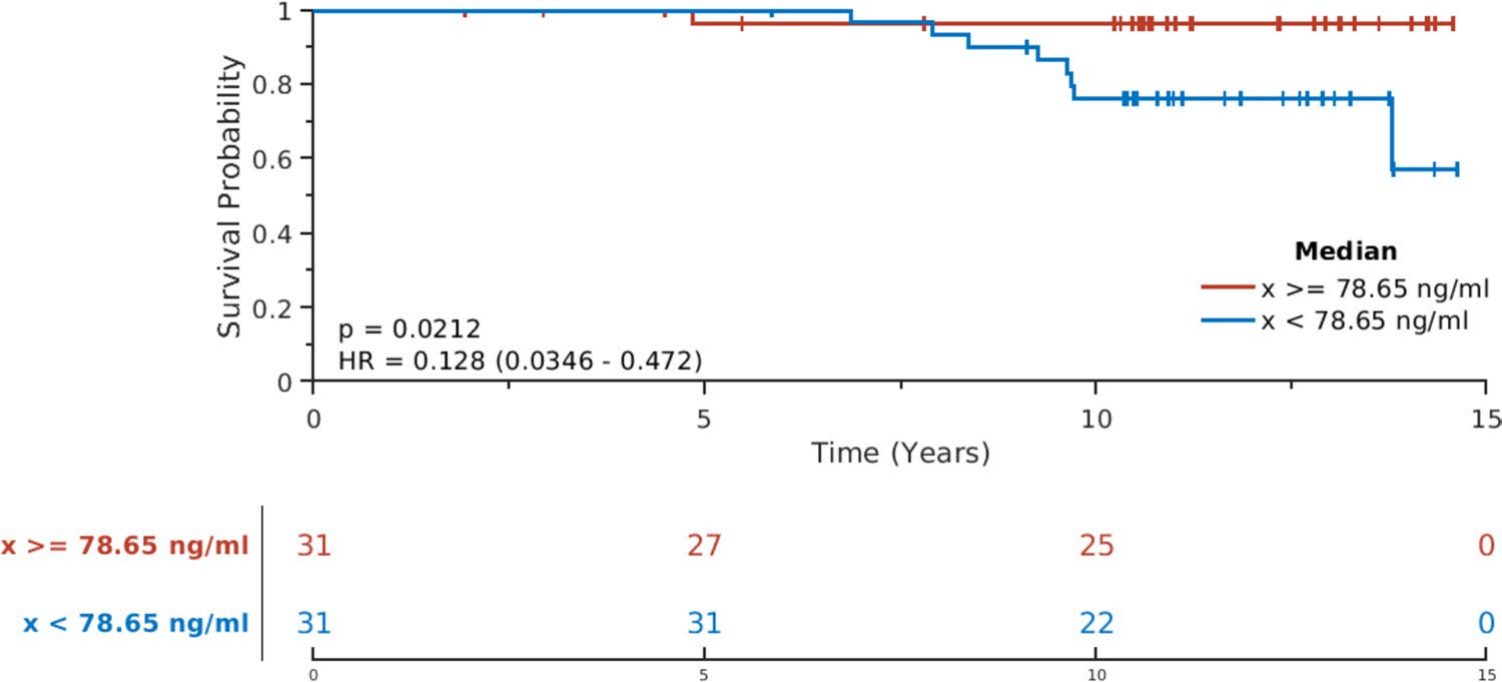
Kaplan–Meier estimates of breast cancer recurrence for patients with high vs. low baseline cortisol levels. Kaplan–Meier plot showed that patients with high (≥ median) baseline cortisol levels had a less recurrence, when comparing with those with low levels (< median). *p* = 0.02, log-rank test; and *HR* = 0.13

Serum steroid hormone concentrations before radiotherapy (TP1) and immediately after radiotherapy (TP2) were able to discriminate between patients with and without recurrent breast cancer with 68.1% and 63.2% accuracy (permutation *p* = 0.02 and 0.03, respectively) (Fig. 1). The two most important steroid hormones for differentiation were cortisol and cortisone, which were present at lower concentrations in patients with breast cancer recurrence. In addition, LMM showed that baseline concentrations of cortisol were lower in patients experiencing breast cancer recurrence than in patients without recurrence (adjusted *p* < 0.05, Fig. 2A). While there was a decrease in cortisol concentration at TP4-TP6 in relapse-free patients, LMM showed that cortisol concentration increased at TP3-TP6 in patients with breast cancer recurrence (adjusted *p* < 0.05 and 0.01 for TP3 and TP6, respectively, Fig. 2A). Similarly, compared to baseline (TP1), cortisone concentrations in patients who did not relapse decreased at TP6 (adjusted *p* ˂0.01), but increased at TP4 and TP6 in patients who did relapse (adjusted *p* = 0.04 and < 0.05, respectively, Fig. 2B). There were no significant changes in the concentrations of other steroid hormones. Kaplan–Meier analysis showed that patients with high baseline concentrations of cortisol (≥ median, 78.65 ng/ml) were significantly less likely to relapse than patients with low concentrations (< median, 78.65 ng/ml) (*p* = 0.02, log-rank-test, *HR* = 0.13, Fig. 3). No significant difference in recurrence rate was found between patients with high and low baseline cortisone levels (*p* = 0.41, log-rank test; *HR* = 0.56, Supplementary Fig. 1).

### Associations of serum steroid hormone levels with fatigue

**Fig. 4:**
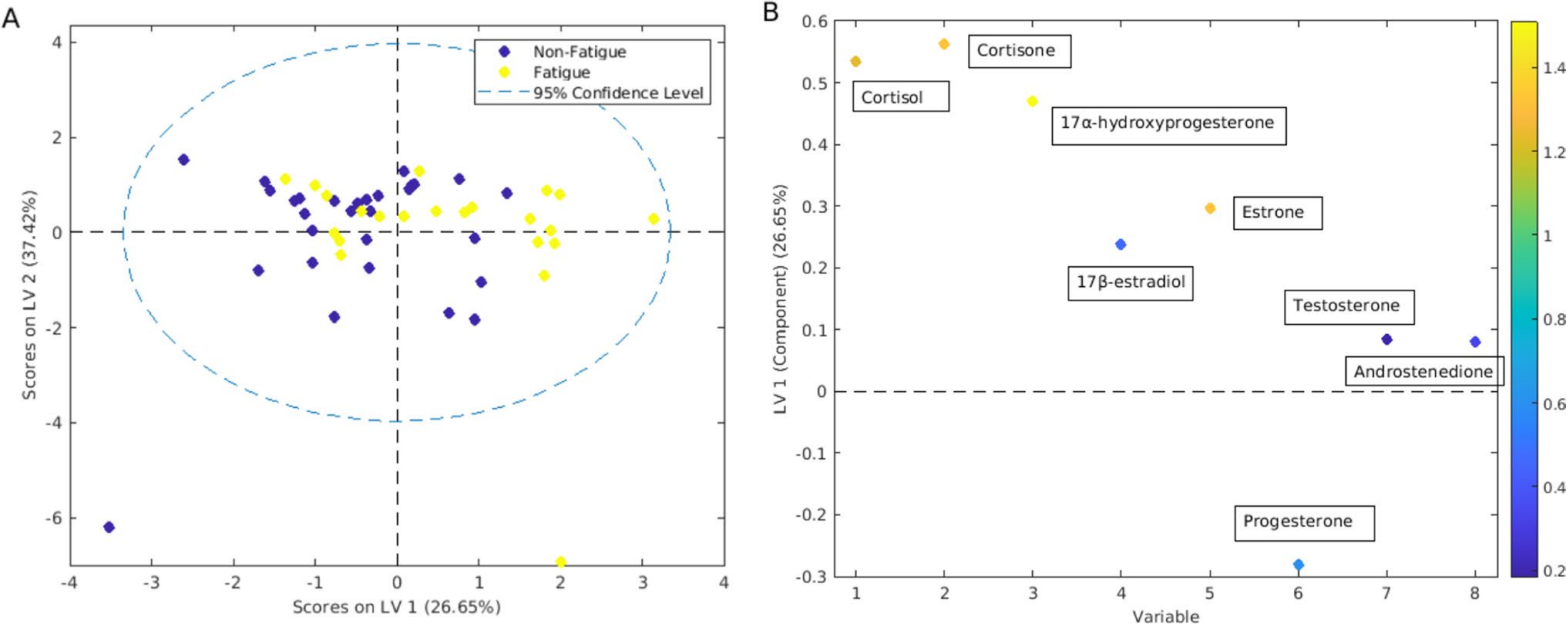
Scores and loading plots from PLS-DA for prediction of fatigue by steroid hormones**.** Both serum steroid hormone levels and treatment-related fatigue were measured at TP2 (*n* = 51). Prediction sensitivity: 67.1%, specificity: 58.4%, accuracy = 62.7%, permutation *p* = 0.031

PLS-DA showed that serum concentrations of steroid hormones at TP2 were significantly different between patients with and without clinically relevant physical fatigue (sensitivity, specificity, and accuracy of 61.7%, 45.5%, and 58.4%, respectively, *p* = 0.03, Fig. 4). The main steroid hormones for discrimination were cortisol, cortisone, 17α-hydroxyprogesterone, and estrone. Baseline steroid hormone concentrations (TP1) could not predict whether patients would experience clinically relevant physical fatigue at 1 year or 7–12 years after treatment (TP5 or TP6). Similarly, LMM analysis showed that changes in serum steroid hormone levels from TP1 to TP2-TP6 did not differ significantly between patients with and without clinically relevant physical fatigue at TP5 or TP6. In addition, no significant association between steroid hormone levels and mental fatigue (reduced cognitive functioning) was observed at any time point.

### Changes of serum steroid hormone concentrations in patients with tamoxifen/aromatase inhibitors or aromatase inhibitor treatment

In patients treated with aromatase inhibitors (*n* = 10), serum concentrations of cortisol, cortisone, and 17α-hydroxyprogesterone were higher at TP1 than at the later time points TP2-6 (Wilcoxon signed-rank tests, adjusted *p* ˂ 0.05, Supplementary Fig.2 A–C and Supplementary Table 1). In addition, serum levels of 17β-estradiol, estrone, and androstenedione were also higher in TP1 compared with TP2 (adjusted *p* = 0.03, = 0.04, and < 0.05, respectively, Supplementary Figs. 2D, 2E and 2H). At TP2-5, levels of 17β-estradiol and estrone were below the lower limit of quantification (LLOQ) in most patients treated with aromatase inhibitors, whereas one patient who later developed recurrence had quantifiable estrone levels at TP2 and TP3 (Supplementary Fig. 2D–E). Testosterone and progesterone levels were not different between TP1 and later time points (adjusted *p* > 0.05, Supplementary Fig. 2F–G). In patients with sequential use of tamoxifen followed by aromatase inhibitors (tamoxifen starting during radiotherapy, *n* = 53) and the entire patient cohort (*n* = 66), there were no significant changes in steroid hormone levels between TP1 and later time points TP2-6, nor between two consecutive time points (Supplementary Fig. 2I–P and Supplementary Fig. 3, respectively).

## Discussion

We demonstrated that serum concentrations of steroid hormones measured before and immediately after radiotherapy were able to differentiate patients experiencing recurrent breast cancer from those who remained free of recurrence during follow-up. After radiation and endocrine treatment, changes in cortisol and cortisone levels differed significantly, with levels increasing in patients experiencing recurrence and decreasing in those free of recurrence (Supplementary Fig. 4). Immediately after radiotherapy, steroid hormone levels could differentiate patients with and without physical fatigue.

We found that patients with high baseline cortisol levels (≥ median) were less likely to relapse than patients with low baseline cortisol levels (< median). Patients who relapsed also had higher BMI compared with those without relapse. The relationship between BMI and cortisol levels has been studied extensively, but results remain inconsistent. Some studies report an inverse correlation between cortisol and BMI [29, 30], while others show a direct correlation [31] or a U-shaped correlation [32]. In our study, after adjusting for BMI as one of the possible risk factors in LMM (Fig. 3), we observed differences in baseline cortisol levels and longitudinal changes in cortisol and cortisone between the patient groups with and without recurrence. This observation suggests that, independent of BMI, baseline cortisol and cortisone levels and their changes during follow-up are predictive of recurrence in the long term. The synthesis and secretion of cortisol are regulated by the hypothalamic-pituitary-adrenal (HPA) axis which in healthy individuals follows a diurnal rhythm where the levels are highest in the morning, decrease throughout the day, and are lowest at night. However, under chronic inflammatory and/or stressful conditions, such as breast cancer, HPA feedback mechanisms can be impaired, resulting in a dysregulated (e.g., “flattened”) diurnal cortisol rhythm [33, 34]. Flattened diurnal cortisol curves have been shown to be associated with shortened survival in patients with metastatic breast cancer [11]. In addition, increased serum cortisol levels have been observed in patients with primary and metastatic breast cancer compared with healthy controls [35]. The basic mechanisms explaining how lower baseline concentrations of cortisol may increase the risk of recurrence remain unclear. However, it is known that cortisol must bind to the glucocorticoid receptor (GR) to activate its signaling pathway. In a retrospective meta-analysis of gene expression in primary breast tumors, Pan and colleagues showed that high expression of GR was significantly correlated with better outcome in ER positive breast tumors, but not in ER negative tumors [36, 37]. This difference may be due to an interaction of GR and ER. We suggest that activated GR signaling pathways may explain the positive association between cortisol levels and better clinical outcomes in ER positive breast cancer patients in our cohort.

In our study, sex hormones (including 17β-estradiol, estrone, testosterone, androstenedione, and progesterone) measured both before and after radiation and endocrine treatment failed to predict breast cancer recurrence. Previous studies examining the association between circulating sex hormones and breast cancer survival have been limited and conflicting. Similar to our findings, a multiethnic prospective cohort study (*n* = 358) showed no association between post-diagnostic levels of 17β-estradiol, estrone, or testosterone and breast cancer-specific and all-cause mortality in postmenopausal breast cancer patients [38]. In contrast, several sex hormones have been reported to be associated with the risk of breast cancer recurrence or survival. For example, both pre- and post-diagnostic circulating 17β-estradiol concentrations, but not testosterone and sex hormone-binding globulin concentrations, have been shown to be associated with poorer survival in postmenopausal breast cancer patients and risk of recurrence in patients with early stage breast cancer [12, 39]. In addition, high testosterone levels have been associated with poorer survival in normal weight/overweight breast cancer patients but with better survival in obese patients [13, 40, 41]. These conflicting results may be due to the different study designs and patient cohorts. Compared with pre-diagnostic levels, steroid hormone levels at the time of diagnosis or after diagnosis may better reflect the hormonal milieu in the breast that can lead to treatment resistance and recurrence [12]. In patients treated with aromatase inhibitors, we found that estrone levels were quantifiable in patients with recurrence within the first year after initiation of endocrine treatment, although 17β-estradiol levels were below LLOQ (Supplementary Fig. 1). In agreement with our findings, Mattson and colleagues recently reported that monitoring serum estrogen levels (17β-estradiol) in patients during prolonged adjuvant letrozole treatment after five years of tamoxifen can be used to predict endocrine failure by a sensitive and specific LC–MS/MS method [42]. Both our study and Mattson’s study suggest that failure of estrogen suppression may be a potential biomarker for predicting recurrence in patients treated with aromatase inhibitors [42].

Although the pathophysiology of cancer-related fatigue remains to be elucidated, evidence has accumulated that dysregulations of the HPA axis (or inadequate glucocorticoid signaling) are involved [17]. Reduced serum cortisol levels or decreased expression of transcripts with response elements for glucocorticoids have been observed in fatigued breast cancer survivors [43, 44]. In addition, several studies have found that fatigue in breast cancer patients is associated with diurnal cortisol dysregulation [18, 45]. In contrast, serum levels of cortisol and steroid hormones (such as 17β-estradiol and dehydroepiandrosterone) were not associated with the development of fatigue in breast cancer patients receiving adjuvant chemotherapy [46]. The mechanism responsible for fatigue may vary among breast cancer therapies. In our cohort, baseline serum steroid hormone levels did not predict physical and mental fatigue during follow-up. However, we found that levels measured immediately after radiotherapy (mainly cortisol, cortisone, 17α-hydroxyprogesterone, and estrone) differed in patients with and without physical fatigue at the same time point. This may suggest that serum cortisol levels reflect HPA function during sampling (with radiotherapy as a stressor) but not 1 year or 7–12 years later.

Our study has limitations. First, because of the small patient cohort, it was not possible to compare the role of steroid hormone levels between different endocrine treatment options. Second, all subjects included in this study were postmenopausal, and thus, we cannot examine the influence of menopausal status on changes in steroid hormone levels. Third, it should be noted that our baseline samples were taken before the start of radiotherapy and approximately 1–4 months after surgery. Therefore, steroid hormone levels could be affected by the extent of surgery or the difference in time since surgery. Finally, serum samples were collected during normal working hours (weekdays from 8 am to 4 pm), so diurnal cortisol rhythms cannot be taken into account. To validate and extend our findings, a future study should be conducted with a larger cohort of patients, including both premenopausal and postmenopausal patients, and sampling at a fixed time of day to strengthen our preliminary results.

In conclusion, breast cancer patients who had lower serum cortisol concentrations before radiotherapy were more likely to experience breast cancer recurrence. During follow-up, cortisol and cortisone levels increased in patients who experienced recurrence, whereas they decreased in relapse-free patients. Our results suggest cortisol and cortisone levels in serum samples as potential biomarkers for risk assessment and decision making in early hormone receptor-positive breast cancer in the future.

### Supplementary Information

Below is the link to the electronic supplementary material.

Supplementary file1 (PPTX 367 KB)

Supplementary file2 (DOCX 44 KB)

Supplementary file3 (DOCX 13 KB)

## Data Availability

The data that support the findings of this study are not openly available due to sensitivity of human data but are available from the corresponding author upon request.

## Abbreviations

**ER**: Estrogen receptor
**PR**: Progesterone receptor
**GR**: Glucocorticoid receptor
**HPA**: Hypothalamic-pituitary-adrenal
**LC****–****MS/MS**: Liquid chromatography–tandem mass spectrometry
**GC****–****MS**: Gas chromatography–mass spectrometry
**LLOQ**: Low limit of quantification
**PLS-DA**: Partial least squares discriminant analysis
**LMM**: Linear mixed model
**EORTC**: The European Organization for Research and Treatment of Cancer
**QLQ-C30**: Quality of Life Questionnaire 30
**BMI**: Body mass index
