# Supplementary material for: Association of serum cortisol and cortisone levels and risk of recurrence after endocrine treatment in breast cancer
Source: Clin Exp Med. 2023 Jul 3;23(7):3883–93. doi: 10.1007/s10238-023-01109-x (PMC10618334; doi:10.1007/s10238-023-01109-x)
Supplement: Supplementary file 1 — Supplementary file1 (PPTX 367 KB) [file 10238_2023_1109_MOESM1_ESM.pptx]

## Slide 1
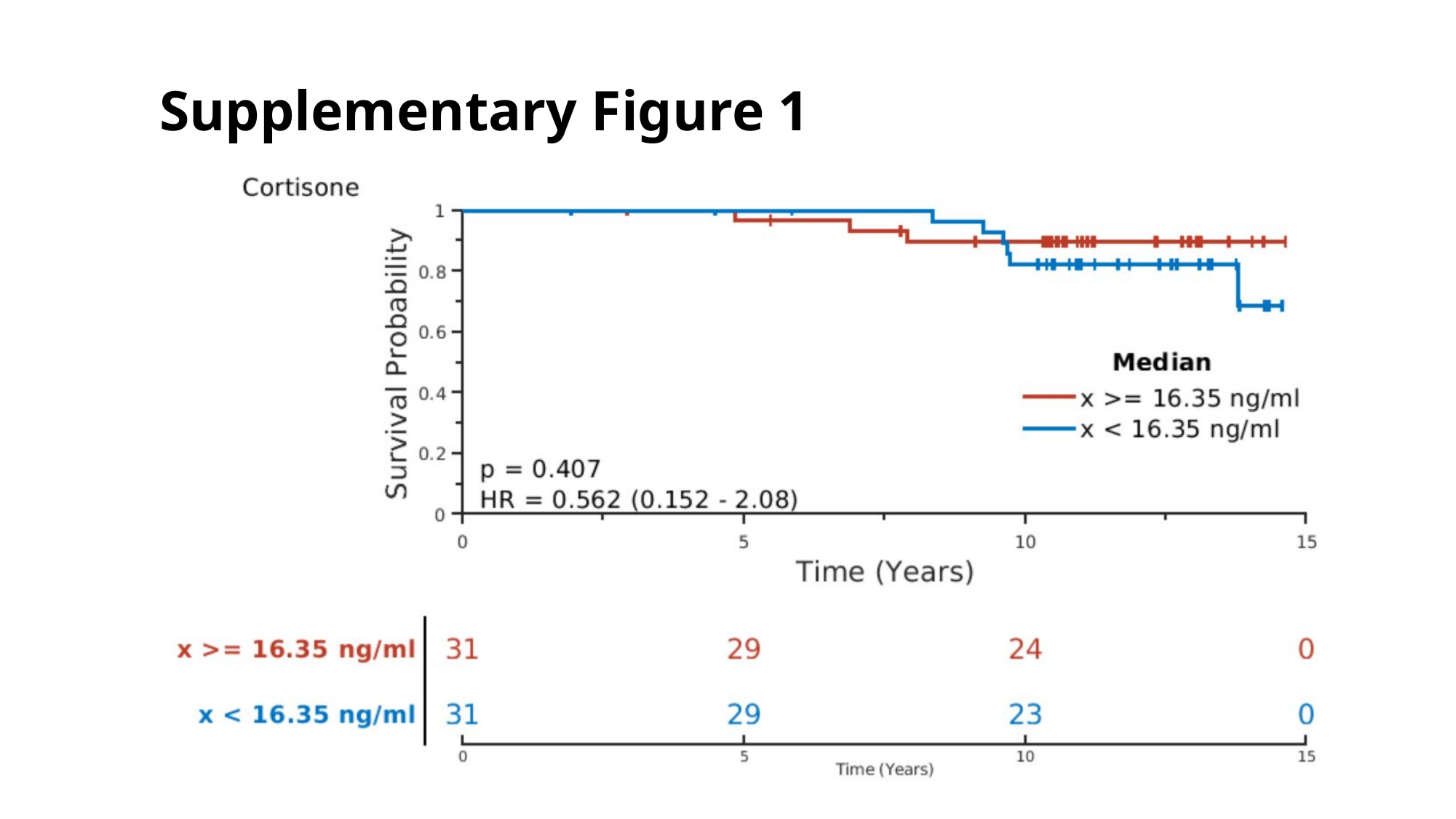

# Supplementary Figure 1

## Slide 2
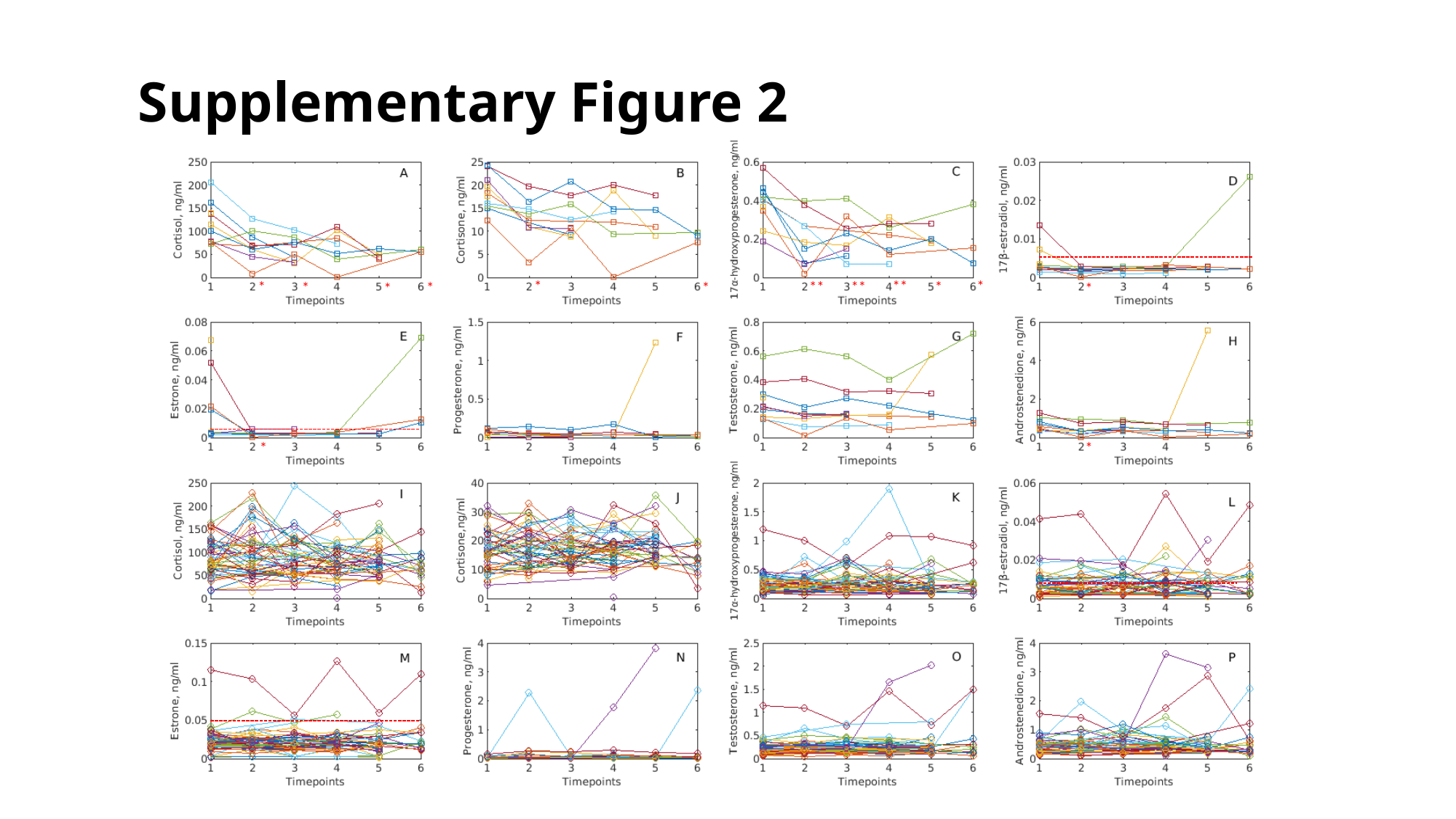

# Supplementary Figure 2
**
*
*
**
*
**
*
*
*
*
*
*
*
*

## Slide 3
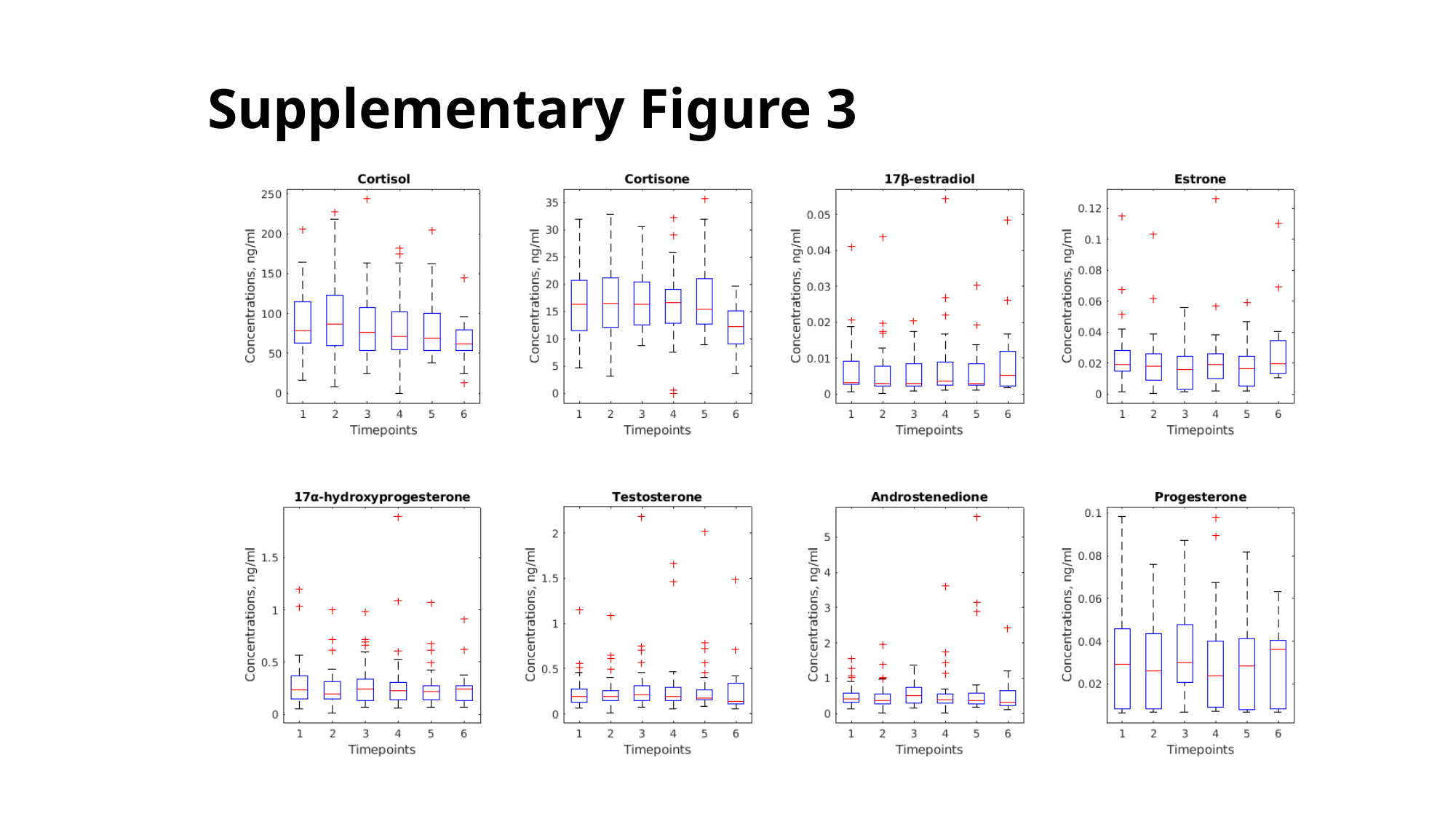

# Supplementary Figure 3

## Slide 4
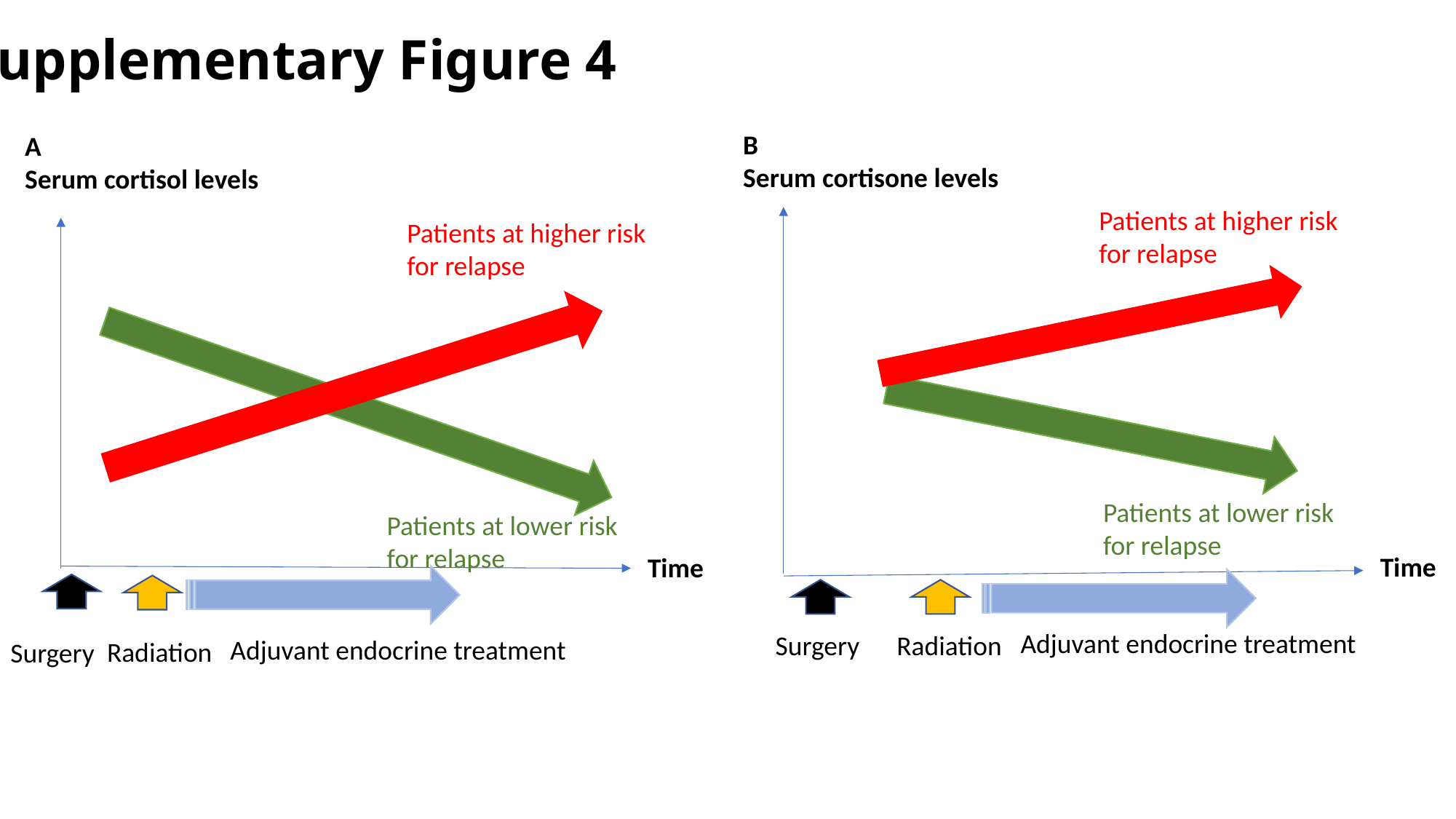

Supplementary Figure 4
B
Serum cortisone levels
A
Serum cortisol levels
Patients at higher risk
for relapse
Patients at higher risk
for relapse
Patients at lower risk
for relapse
Patients at lower risk
for relapse
Time
Time
Adjuvant endocrine treatment
Surgery
Radiation
Adjuvant endocrine treatment
Radiation
Surgery
