# Supplementary material for: Association of serum cortisol and cortisone levels and risk of recurrence after endocrine treatment in breast cancer
Source: Clin Exp Med. 2023 Jul 3;23(7):3883–93. doi: 10.1007/s10238-023-01109-x (PMC10618334; doi:10.1007/s10238-023-01109-x)
Supplement: Supplementary file 2 — Supplementary file2 (DOCX 44 KB) [file 10238_2023_1109_MOESM2_ESM.docx]

| **Supplementary Table 1 Adjusted p-values for Wilcoxon signed-rank tests when compared between TP1* and TP2-6** | | | | | | | | | | |
| --- | --- | --- | --- | --- | --- | --- | --- | --- | --- | --- |
| Patient groups | TP | Endocrine treatment  when sampling | Cortisol | Cortisone | 17α-hydroxy  Progesterone | 17β-estradiol | Estrone | Progest  erone | Testosterone | Androstene  -dione |
| AI | TP2 | AI | 0.03 | 0.03 | <0.01 | 0.03 | 0.04 | 0.24 | 0.45 | <0.05 |
|  | TP3 | AI | 0.03 | 0.07 | <0.01 | 0.07 | 0.09 | 0.42 | 0.70 | 0.70 |
|  | TP4 | AI | 0.11 | 0.11 | <0.01 | 0.15 | 0.11 | 0.81 | 0.69 | 0.12 |
|  | TP5 | AI | 0.01 | 0.14 | 0.01 | 0.49 | 0.21 | 0.87 | 0.87 | 0.87 |
|  | TP6 | no | 0.02 | 0.02 | 0.02 | 0.94 | 0.46 | 0.54 | 0.54 | 0.43 |
| Tamoxifen  /AI | TP2 | Tamoxifen | 0.68 | 0.68 | 0.86 | 0.86 | 0.99 | 0.86 | 0.86 | 0.86 |
|  | TP3 | Tamoxifen | 0.64 | 0.58 | 0.58 | 0.95 | 0.71 | 0.64 | 0.58 | 0.58 |
|  | TP4 | Tamoxifen | 1.00 | 1.00 | 1.00 | 1.00 | 1.00 | 1.00 | 1.00 | 1.00 |
|  | TP5 | Tamoxifen | 0.97 | 0.81 | 0.97 | 0.97 | 0.81 | 0.81 | 0.81 | 0.81 |
|  | TP6 | no | 0.91 | 0.91 | 0.91 | 0.91 | 0.91 | 0.91 | 0.91 | 0.91 |
| AI, Aromatase inhibitor treatment for 5 years, starting during radiotherapy. | | | | | | | | | | |
| Tamoxifen/AI, sequceing use of Tamoxifen followed by AI. Tamoxifen starting during radiotherapy. | | | | | | | | | | |
| TP, time point. *, TP1, before starting radiotherapy and endocrine treatment. | | | | | | | | | | |

| **Supplementary Table 2. Clinical information of patints experiencing recurrent breast cancer** | | | | | | | | | | | |
| --- | --- | --- | --- | --- | --- | --- | --- | --- | --- | --- | --- |
| Patient | Relapse or death after diagnose (years) | Age at diagnose | BMI | Grade | Nodal spread | Tumour size | Stage | HER2 | ER | PgR | Endocrine treatment |
| 1 | 9.6 | 68 | 26.8 | 3 | N0 | T1 | I | negative | postive | positive | Tamoxifen/AI |
| 2 | 13.8 | 64 | 29.4 | 2 | N1 | T1 | IIA | negative | postive | positive | Tamoxifen/AI |
| 3 | 9.3 | 63 | 30.7 | 2 | N0 | T1 | I | negative | postive | positive | Tamoxifen/AI |
| 4 | 9.7 | 67 | 34.4 | 2 | N1 | T2 | IIB | negative | postive | positive | Tamoxifen/AI |
| 5 | 8.4 | 60 | 28.8 | 2 | N0 | T1 | I | negative | postive | negative | Tamoxifen/AI |
| 6 | 9.7 | 65 | 26.5 | 2 | N1 | T2 | IIB | negative | postive | positive | Tamoxifen/AI |
| 7 | 8.8 | 89 | 24.7 | 2 | N0 | T1 | I | negative | postive | negative | Tamoxifen/AI |
| 8 | 4.8 | 55 | 25.9 | 2 | N0 | T1 | I | negative | postive | positive | Tamoxifen/AI |
| 9 | 6.9 | 71 | 25.1 | 3 | N1 | T1 | IIA | positive | postive | positive | Tamoxifen/AI |
| 10 | 7.9 | 69 | 34.0 | 3 | N2 | T2 | IIIA | positive | postive | positive | AI |
| Tamoxifen/AI, sequceing use of Tamoxifen followed by AI. Tamoxifen starting during radiotherapy. | | | | | | | | | | | |
| AI, Aromatase inhibitor treatment for 5 years. | | | | | | | | | | | |
| HER2, human epidermal growth factor receptor 2. | | | | | | | | | | | |
| ER, estrogen receptor. | | | | | | | | | | | |
| PgR, progesterone receptor.  Tumer size: T1, <2 cm; T2, 2–5 cm. | | | | | | | | | | | |
