# Supplementary material for: Association of serum cortisol and cortisone levels and risk of recurrence after endocrine treatment in breast cancer
Source: Clin Exp Med. 2023 Jul 3;23(7):3883–93. doi: 10.1007/s10238-023-01109-x (PMC10618334; doi:10.1007/s10238-023-01109-x)
Supplement: Supplementary file 3 — Supplementary file3 (DOCX 13 KB) [file 10238_2023_1109_MOESM3_ESM.docx]

**Titles and Legends for the SUPPLEMENTARY figures**

**Supplementary Figure 1**

Title: Kaplan-Meier estimates of breast cancer recurrence for patients with high vs. low baseline cortisone levels.

Legend: Kaplan-Meier plot showed that breast cancer recurrence was not significantly different between patients with high (≥median) and low (< median) baseline cortisone levels. p = 0.02, log-rank test; and HR= 0.13. p =0.41, log-rank test; and HR= 0.56.

**Supplementary Figure 2**

Title: Serum levels of steroid hormones cross all timepoints in patients with different endocrine treatments

Legend: A-H: Patients received aromatase inhibitors (n=10). Concentrations of cortisol, cortisone, 17β-estradiol, estrone, 17α-hydroxyprogesterone, and androstenedione, at TP1 were higher compared to later time points TP2-6. *, adjusted p <0.05; **, adjusted p <0.01; Wilcoxon signed-rank tests I-P: Patients treated with sequencing use of tamoxifen followed by aromatase inhibitors (n=53, starting tamoxifen during radiotherapy). No significant changes in steroid hormone levels were found between TP1 and later time points TP2-6 (Wilcoxon signed-rank tests). The LOQs (0.005 ng/ml) for both estrone and 17β-estradiol was marked as red dash lines.

**Supplementary Figure 3**

Title: Boxplots of serum steroid hormone concentrations across all timepoints for the full patient cohort

Legend: No significant changes in steroid hormone levels were found between TP1 and later time points TP2-6, nor between two consecutive time points (Wilcoxon signed-rank test). The red + marker symbol represents outliers. For progesterone, concentrations that exceeded 10th and 90th percentiles were excluded.

**Supplementary Figure 4**

Title: Summary of overall results: Cortisol and cortisone as potential biomarkers for risk of recurrence

Legend: Serum cortisol levels measured after surgery and before radiotherapy were high in patients at low risk of recurrence, although cortisone levels were not different between patients at high and low risk of recurrence. Both serum cortisol and cortisone levels decreased over time in patients at low risk of recurrence and increased in patients at high risk.
